# Reducing scan time for calibration of through-time radial GRAPPA using PCA coil compression

**DOI:** 10.1186/1532-429X-17-S1-Q41

**Published:** 2015-02-03

**Authors:** Jesse I Hamilton, Caroline Zuchold, Nicole Seiberlich

**Affiliations:** 1 Biomedical Engineering, Case Western Reserve University, Cleveland, OH, USA; 2 Hathaway Brown, Shaker Heights, OH, USA

## Background

Although using more coils may improve parallel imaging performance, it produces a burden on the amount of calibration data required for autocalibrating methods. This work investigates the use of principal component analysis (PCA) to project the original set of coils onto a smaller set of virtual coils to reduce calibration scan time for through-time radial GRAPPA [Seiberlich, et al. Magn Reson Med. 2011; 65(2):492-505].

## Methods

Simulations were performed using a randomly moving Shepp-Logan phantom and a simulated 24-channel array. Calibration data (144 projection, 80 frames, 128x128 matrix) were generated along a fully-sampled radial trajectory, and an additional 40 frames were undersampled to 24, 16, and 12 projections. Through-time radial GRAPPA reconstruction was performed with a 4x1 (read x projection) k-space segment size and varying numbers of calibration frames. The reconstructions were repeated after PCA coil compression that captured 93%, 91%, 87% and 81% of the variation in the original coil maps (12, 9, 6, and 4 virtual coils). In vivo cardiac data were also collected in one healthy volunteer in this IRB approved, HIPAA compliant study using a 30 channel receiver array on a 3T scanner. Both calibration (144 projections, 160 frames, 68s) and undersampled (24, 16, and 12 projections) data were collected using a radial bSSFP sequence with 128x128 matrix and TR/TE=2.94/1.47ms. Data were collected in short-axis and 4-chamber orientations to demonstrate how differences in coil geometry affect the acceptable amount of PCA coil compression when combined with parallel imaging.

## Results

**Figure 1:**
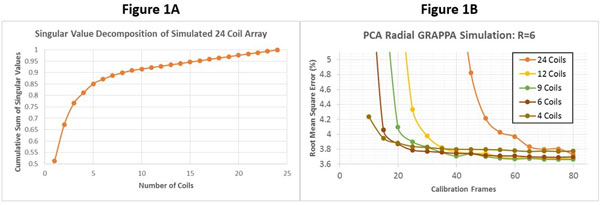
A: The fraction of variation in the original 24-channel coil map is displayed as a function of coil number. Figure 1B: RMSE is plotted against the number of calibration frames for a through-time radial GRAPPA reconstruction at an acceleration of R=6 after different amounts of coil compression.

**Figure 2:**
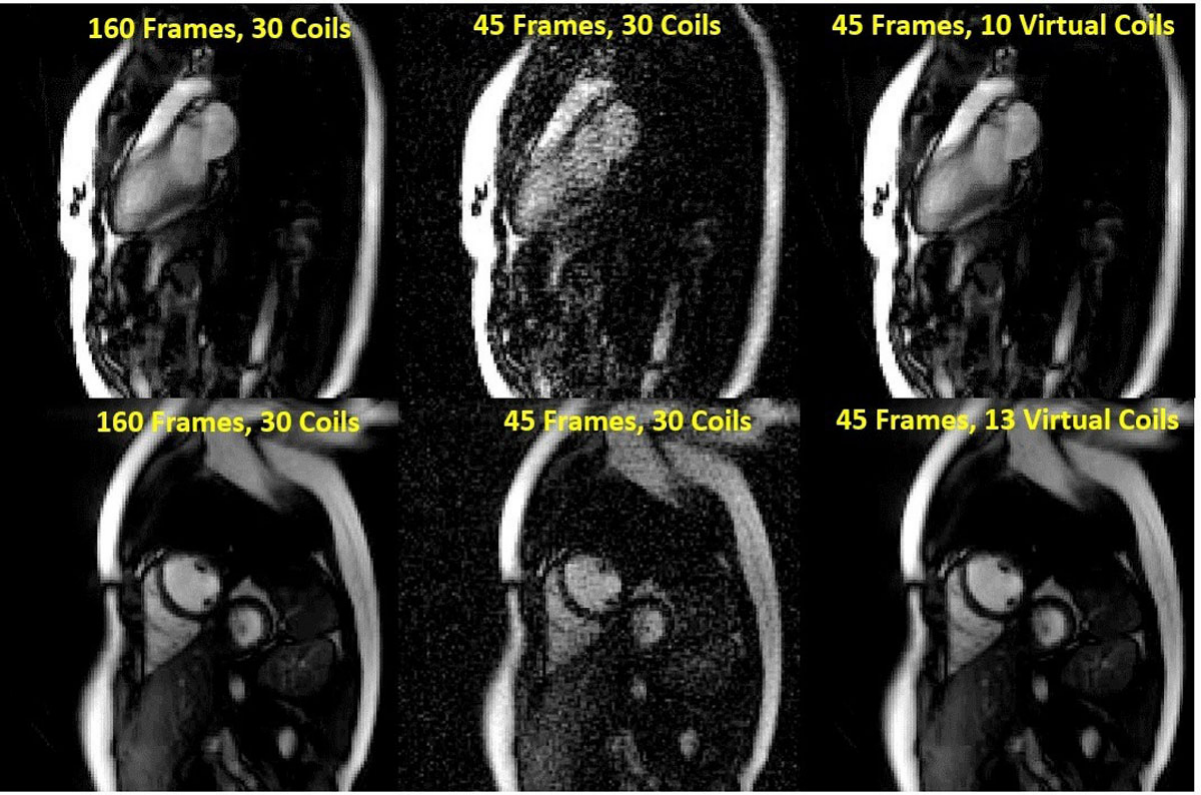
Real-time, free-breathing cardiac images were reconstructed with through-time radial GRAPPA in long axis and short axis orientations using different numbers of calibration frames and virtual coils.

As shown by the cumulative sum of the singular values in Figure 1A, the simulated coil sensitivities are redundant with 95% of the variation captured by 15 coils. Figure 1B depicts the RMSE for through-time radial GRAPPA using different numbers of calibration frames and virtual coils with an acceleration factor of R=6; similar trends were observed at R=9 and R=12. The RMSE increases sharply as the number of calibration frames decreases from 80 to 36, where the GRAPPA weight equation is exactly determined. However, the RMSE with 9 virtual coils, or 90% of the variation in the original sensitivity maps, remains below 4% even when 25 calibration frames are used. In vivo cardiac images in long and short axis orientations are shown in Figure 2. When using all 30 coils, the reconstruction exhibits substantial noise enhancement as the calibration frames are decreased from 160 to 45, where the GRAPPA weight equation is exactly determined. Based on simulation results, the datasets were compressed to retain 90% of the original coil sensitivity variation, after which using 45 calibration frames appears qualitatively identical to the 160 frame/30 coil reconstruction.

## Conclusions

PCA coil compression can be used to reduce calibration scan time for through-time radial GRAPPA. This technique may be especially useful for interventional imaging or 3D parallel imaging.

## Funding

NIH/NIBIB R00EB011527 and Siemens Medical Solutions.

